# Early predictors of intensive care unit admission among COVID-19 patients in Qatar

**DOI:** 10.3389/fpubh.2024.1278046

**Published:** 2024-03-20

**Authors:** Safae Abuyousef, Shaikha Alnaimi, Nabil E. Omar, Reem Elajez, Eman Elmekaty, Eiman Abdelfattah-Arafa, Raja Barazi, Rola Ghasoub, Ala Rahhal, Fatima Hamou, Maha Al-Amri, Ahmed Karawia, Fatima Ajaj, Raja Alkhawaja, Ahmed Kardousha, Ahmed Awaisu, Adel Abou-Ali, Mohamad Khatib, Mohammed Aboukamar, Moza Al-Hail

**Affiliations:** ^1^Department of Pharmacy, Heart Hospital, Hamad Medical Corporation, Doha, Qatar; ^2^Department of Pharmacy, Hamad Bin Khalifa Medical City, Hamad Medical Corporation, Doha, Qatar; ^3^Department of Pharmacy, National Centre for Cancer Care and Research, Hamad Medical Corporation, Doha, Qatar; ^4^Health Sciences Program, Clinical and Population Health Research, College of Pharmacy, Qatar University, Doha, Qatar; ^5^Department of Pharmacy, Hamad General Hospital, Hamad Medical Corporation, Doha, Qatar; ^6^Department of Pharmacy, Communicable Diseases Center, Hamad Medical Corporation, Doha, Qatar; ^7^Department of Pharmacy, Al-Khor Hospital, Hamad Medical Corporation, Doha, Qatar; ^8^Department of Pharmacy, Al Wakra Hospital, Hamad Medical Corporation, Doha, Qatar; ^9^Department of Pharmacy, Rumailah Hospital, Hamad Medical Corporation, Doha, Qatar; ^10^Department of Pharmacy, Home Health Care, Hamad Medical Corporation, Doha, Qatar; ^11^College of Pharmacy, QU Health, Qatar University, Doha, Qatar; ^12^Astellas Pharma Global Development, Inc., Northbrook, IL, United States; ^13^Department of Critical Care, Hamad General Hospital, Hamad Medical Corporation, Doha, Qatar; ^14^Department of Infectious Disease, Communicable Diseases Center, Hamad Medical Corporation, Doha, Qatar; ^15^Department of Pharmacy, Women’s Wellness and Research Center, Hamad Medical Corporation, Doha, Qatar

**Keywords:** COVID-19, predictors, risk factors, intensive care unit, mortality, critically ill patients

## Abstract

**Background:**

COVID-19 is associated with significant morbidity and mortality. This study aimed to explore the early predictors of intensive care unit (ICU) admission among patients with COVID-19.

**Methods:**

This was a case–control study of adult patients with confirmed COVID-19. Cases were defined as patients admitted to ICU during the period February 29–May 29, 2020. For each case enrolled, one control was matched by age and gender.

**Results:**

A total of 1,560 patients with confirmed COVID-19 were included. Each group included 780 patients with a predominant male gender (89.7%) and a median age of 49 years (interquartile range = 18). Predictors independently associated with ICU admission were cardiovascular disease (adjusted odds ratio (aOR) = 1.64, 95% confidence interval (CI): 1.16–2.32, *p* = 0.005), diabetes (aOR = 1.52, 95% CI: 1.08–2.13, *p* = 0.016), obesity (aOR = 1.46, 95% CI: 1.03–2.08, *p* = 0.034), lymphopenia (aOR = 2.69, 95% CI: 1.80–4.02, *p* < 0.001), high AST (aOR = 2.59, 95% CI: 1.53–4.36, *p* < 0.001), high ferritin (aOR = 1.96, 95% CI: 1.40–2.74, *p* < 0.001), high CRP (aOR = 4.09, 95% CI: 2.81–5.96, *p* < 0.001), and dyspnea (aOR = 2.50, 95% CI: 1.77–3.54, *p* < 0.001).

**Conclusion:**

Having cardiovascular disease, diabetes, obesity, lymphopenia, dyspnea, and increased AST, ferritin, and CRP were independent predictors for ICU admission in patients with COVID-19.

## 1 Background

Coronaviruses are a group of viruses that belong to the orthocoronavirinae subfamily (1). This family includes viruses responsible for several past outbreaks, such as the severe acute respiratory syndrome (SARS) and the Middle East Respiratory Syndrome (MERS) caused by SARS-associated coronavirus (SARS-CoV) and the MERS coronavirus (MERS-CoV), respectively (2, 3). The most recent outbreak is the coronavirus disease 2019 (COVID-19) caused by the novel SARS coronavirus 2 (SARS-CoV-2) (4). SARS-CoV-2 was first isolated in Wuhan, China (5) and rapidly spread to become a worldwide pandemic as declared by the World Health Organization (WHO) in March 2020 due to its alarming level of severity and widespread globally (6, 7).

The COVID-19 is associated with a broad spectrum of symptoms ranging from subtle mild symptoms such as fever, cough, and myalgia to severe pneumonia, acute respiratory distress, multi-organ failure, and death (8, 9). The majority of patients fall into the asymptomatic to mild disease category (10–16), while 15.7–26.1% present with severe disease requiring hospitalization and close monitoring (17, 18). Furthermore, an estimated 5–8% of infected patients require intensive care unit (ICU) admission and are at a higher risk of mortality (19–22).

Several studies have reported the risk factors and predictors of poor prognosis and in-hospital death in COVID-19 patients (21–33). The reported risk factors are related to computed tomography (CT) findings (23–25), hematological changes including lymphocyte count and serum ferritin level, as well as other abnormal laboratory findings (23, 26–30). In particular, old age (≥65 years), body mass index (BMI) ≥30 kg/m^2^, and increased procalcitonin are reported as independent risk factors for ICU admission and in-hospital mortality among patients diagnosed with COVID-19 (30–33). There is variability in the predictors of poor prognosis and mortality reported by different studies and different geographical locations. Most studies have assessed risk factors for poor prognosis at a later stage of hospital admission. In contrast, the present study aimed to investigate clinical and laboratory markers present as early as 24 h from admission. One study from the United States has developed a risk scoring system for early identification of rapidly deteriorating patients (34), but no similar study was reported from the Middle Eastern perspective. Since the virulence of the virus may vary by geographical location (35), there is a need for early identification of risk factors for patients who may require ICU admission to allow for optimal deployment and utilization of healthcare resources in Qatar.

Identification of these risk factors may help clinicians and healthcare authorities triage patients, develop a decision support system, and prioritize high-risk COVID-19 patients. This study’s primary objective was to identify the early demographic, clinical, and laboratory predictors of ICU admission among COVID-19 patients in Qatar. The secondary objectives of the study were to describe the characteristics of the patients admitted to the ICU and to explore the predictors of in-hospital mortality.

## 2 Methods

### 2.1 Study design and setting

A retrospective case–control study involving patients diagnosed with COVID-19 who were admitted to any designated COVID-19 healthcare facility across Hamad Medical Corporation (HMC) in Qatar was conducted. HMC is the leading secondary and tertiary healthcare provider in the State of Qatar. Due to the COVID-19 outbreak and to enable the provision of optimum care for COVID-19 patients, two new dedicated hospitals were commissioned, leading to an increase in the number of non-ICU and ICU beds to 3,469 and 529, respectively. In HMC, a confirmed SARS-CoV-2 infection was based on a positive real-time polymerase chain reaction (RT-PCR) assay of nasopharyngeal and oropharyngeal swab specimens. The study was conducted in accordance with the Declaration of Helsinki, and the protocol was granted expedited approval by the HMC Institutional Review Board at the Medical Research Center with a waiver of informed consent (protocol code MRC-05-025 and approved on 29 April 2020). All data were de-identified, except dates of admission and hospital stay.

### 2.2 Patients and eligibility criteria

Eligible cases were adult patients (>18 years old) with a confirmed diagnosis of SARS-CoV-2 infection, admitted to the ICU during the period February 29–May 29, 2020. On the other hand, control patients were adults with a confirmed diagnosis of SARS-CoV-2 infection who were admitted to an inpatient ward but did not require intensive care. For each case enrolled in the study, one control was matched by age and gender (i.e., case to control ratio: 1: 1). A whole population sampling approach was used, where we included all eligible patients admitted to the ICU and their age-and-gender matched control during the study period.

### 2.3 Data collection procedure

#### 2.3.1 Data source

Data were obtained from the HMC electronic medical records system (CERNER) by a clinical informatics specialist using a built-in analytical tool. After initial extraction, data were verified and variables that were difficult to extract using the built-in tool were manually collected from the electronic medical records. The data obtained included: demographics, admission date, discharge data, comorbidities, medication administration records, social history, laboratory data, vital signs, and pertinent clinical notes.

#### 2.3.2 Variables

The specific variables collected for each case or control patient were: age; gender; region of origin; comorbidities; medications administered; smoking status; BMI; co-infection; other variables of signs and symptoms on and during admission including systolic and diastolic blood pressure, mean arterial pressure, heart rate, respiratory rate, presence of dry cough, productive cough, nausea, vomiting, hemoptysis, fatigue, myalgia, headache, confusion, sore throat, diarrhea, and chest pain; oxygen saturation; white blood cells count; hemoglobin; platelets; absolute neutrophil count; lymphocyte count; serum creatinine; albumin; alanine aminotransferase (ALT); aspartate aminotransferase (AST); C-reactive protein (CRP); lactic acid; creatinine kinase (CK); D-dimer; fibrinogen; prothrombin time; international normalized ratio (INR); lactate dehydrogenase (LDH); procalcitonin; ferritin; N-terminal pro-brain natriuretic peptide (NT Pro-BNP); troponin; and radiographic chest findings.

### 2.4 Statistical analysis

All variables were summarized using appropriate descriptive statistics. Categorical variables were reported as frequencies and percentages, while continuous variables were reported as mean ± standard deviation or median (interquartile range, IQR) based on the data’s normality. To compare between ICU and non-ICU patients, Chi-square or Fisher’s exact tests were used for categorical variables. In contrast, the Student *t*-test or Mann–Whitney *U* test were applied for continuous variables as appropriate.

Univariate logistic regression analysis was conducted for baseline data to explore the risk factors associated with ICU admission. In the multivariate logistic regression, we included variables from univariate analysis with a value of *p* < 0.2 and clinically relevant variables. However, we limited the variables to 10 to avoid overfitting of the model. Thus, obesity, cardiovascular disease (CVD), diabetes, pulmonary disease, cancer, lymphopenia, liver injury with AST >3 times the upper limit normal, high CRP, high ferritin, dyspnea upon admission were included in the multivariate logistic regression model. The results are presented as crude odds ratio (OR) and adjusted odds ratio (aOR) with 95% confidence intervals (CIs). A value of *p* < 0.05 was used for statistical significance. Additionally, a multivariate logistic regression of the same ten variables was conducted to determine the predictors of mortality among COVID-19 patients. Data were analyzed using SPSS v25 (IBM SPSS® Statistics for Windows, version 25.0; IBM Corp, Armonk, NY, USA).

## 3 Results

### 3.1 Baseline demographic and clinical characteristics of the subjects on admission

A total of 1,560 patients were included in the analysis. Among these, 780 patients with a confirmed diagnosis of COVID-19 were admitted to the ICU and represented the case group, while 780 COVID-19 patients who were admitted to the hospital but did not require ICU care represented the control group. Both groups were matched for age and gender.

The baseline demographic and clinical characteristics of the patients on admission are presented in Table 1. The cases and controls were similar with respect to age with median of 49 years (IQR = 18), and gender [proportion of male was 89.7% in both groups]. Most patients in both groups were from Asia [ICU admitted 78.7% and non-ICU admitted 75.6%]. On admission, patients in the ICU group were more likely to have a higher Charlson comorbidity index (CCI) (≥3) [11.9% vs. 6.9%, value of *p* < 0.001], diabetes [44.7% vs. 30.8%, value of *p* < 0.001], CVD [41.4% vs. 31.5%, value of *p* < 0.001], chronic kidney disease [7.6% vs. 2.9%, value of *p* < 0.001], pulmonary disease [6% vs. 3.3%, value of *p* 0.013], immunosuppression [2.6% vs. 0.6%, value of *p* 0.005], and stroke [2.2% vs. 0.8%, value of *p* 0.027]. Furthermore, the ICU admitted group had significantly more obese patients [BMI ≥ 30 kg/m^2^] than the non-ICU admitted group [32.4% vs. 26.7%, value of *p* 0.020].

**Table 1:** Baseline characteristics of patients admitted with COVID-19 infection in Qatar (*N* = 1,560).

Characteristic	ICU admitted (*n* = 780)	Non-ICU admitted (*n* = 780)	*p*-value
Age [years], median (IQR)	49 (18)	49 (18)	1^§^
Male gender, *n* (%)	700 (89.7)	700 (89.7)	1^¶^
Region of origin, *n* (%)			0.020^¶^
Africa	16 (2.1)	9 (1.2)	
Asia	614 (78.7)	590 (75.6)	
Europe	3 (0.3)	4 (0.5)	
Middle East	141 (18.1)	176 (22.6)	
North America	6 (0.8)	0	
Australia	0	1 (0.1)	
Comorbidities, *n* (%)			
Diabetes mellitus	349 (44.7)	240 (30.8)	<0.001^¶^
Cardiovascular diseases (HTN, CAD, HF)	323 (41.4)	246 (31.5)	<0.001^¶^
Hypertension (HTN)	296 (37.9)	234 (30)	<0.001^¶^
Coronary artery disease (CAD)	83 (10.6)	41 (5.3)	<0.001^¶^
Heart Failure (HF)	17 (2.2)	7 (0.9)	0.046^¶^
Chronic kidney disease	59 (7.6)	23 (2.9)	<0.001^¶^
Chronic Liver diseases	6 (0.8)	3 (0.4)	0.326^¶^
Pulmonary diseases	47 (6.0)	26 (3.3)	0.013^¶^
Peripheral Vascular Disease	6 (0.8)	12 (1.5)	0.163^¶^
Immunosuppression	20 (2.6)	5 (0.6)	0.005^¶^
Stroke	17 (2.2)	6 (0.8)	0.027^¶^
Cancer	20 (2.6)	6 (0.8)	0.006^¶^
Charlson score, median (IQR)	1 (2)	1 (2)	<0.001^§^
Smoking status, *n* (%) ^a^ Current smoker Ex-smoker Never smoker	32 (4.1) 40 (5.1) 430 (55.1)	36 (4.6) 27 (3.5) 306 (39.2)	0.181^¶^
Body mass index, median (IQR)	27.43 (6.23)	26.92 (5.42)	0.002^§^
Symptoms at admission, *n* (%) Asymptomatic Dyspnea Fever Dry cough Productive cough Hemoptysis Abdominal Pain Nausea Vomiting Chest pain Fatigue Myalgia Headache Confusion Sore throat Diarrhea Others	24 (3.1) 404 (51.8) 635 (81.4) 481 (61.7) 87 (11.2) 10 (1.3) 45 (5.8) 43 (5.5) 69 (8.8) 98 (12.6) 102 (13.1) 186 (23.8) 74 (9.5) 13 (1.7) 105 (13.5) 48 (6.2) 31 (4.0)	162 (20.8) 134 (17.2) 507 (65) 440 (56.4) 23 (2.9) 3 (0.4) 20 (2.6) 14 (1.8) 27 (3.5) 33 (4.2) 37 (4.7) 161 (20.6) 77 (9.9) 5 (0.6) 126 (16.2) 28 (3.6) 25 (3.2)	<0.001^¶^ < 0.001^¶^ < 0.001^¶^ 0.035^¶^ < 0.001^¶^ 0.066^¶^ 0.002^¶^ < 0.001^¶^ < 0.001^¶^ < 0.001^¶^ < 0.001^¶^ 0.128^¶^ 0.797^¶^ 0.068^¶^ 0.135^¶^ 0.020^¶^ 0.414^¶^
Duration from symptoms to hospital admission [days], median (IQR)	4 (4)	4 (5)	0.398^§^
Duration from symptoms to ICU admission [days], median (IQR)	6 (5)	NA	
Vital signs at admission, median (IQR) Systolic blood pressure [mmHg] Diastolic blood pressure [mmHg] Mean arterial pressure [mmHg] Heart rate [bpm] Temperature [^0^C] Respiratory rate [rate/min] Oxygen Saturation [%]	125 (23) 76 (14) 89 (17) 97 (24) 37.5 (1.6) 23 (12) 95 (6)	130 (22) 80 (14) 97.17 (15) 91 (22) 37.2 (1.3) 18 (2) 98 (2)	<0.001^§^ <0.001^§^ <0.001^§^ <0.001^§^ <0.001^§^ <0.001^§^ <0.001^§^

^§^ *p*-value obtained using Mann–Whitney test; ^¶^*p*-value obtained using Chi-square test; ICU, intensive care unit; NA, Not applicable; ^a^ no documentation on smoking status (278 in ICU and 411 in non ICU).

Differences were also observed between the two groups in terms of COVID-19-related clinical presentations on admission. The non-ICU admitted group had more asymptomatic patients than the ICU admitted group [20.8% vs. 3.1%, respectively; value of *p* < 0.001]. On the other hand, dyspnea, fever, dry and productive cough, gastrointestinal symptoms, chest pain, and were more prevalent in the ICU admitted group than the non-ICU admitted group (Table 1).

Similarly, abnormalities in vital signs on admission were more prevalent in the ICU group compared to the non-ICU group, including hypotension, tachycardia, tachypnea, and higher temperature as shown in Table 1.

The differences in the laboratory and radiological findings between the two groups were also significant (Table 2). Leukocytosis, neutrophilia, lymphopenia, anemia, higher serum creatinine, and liver transaminase were more frequent in ICU admitted patients on admission. Significant increase in D-Dimer [median (IQR) 0.87 (1.4) mg/L vs. 0.56 (0.7) mg/L, value of *p* < 0.001], NT-Pro-BNP [median (IQR) 277.8 (987.7) pg./ml vs. 81 (732.2) pg./ml, value of *p* < 0.001], Troponin-T HS [median (IQR) 13 (31) ng/L vs. 8 (6) ng/L, value of *p* < 0.001], and inflammatory and infection markers including CRP, procalcitonin, LDH, ferritin and CK each with (value of *p* < 0.001) were observed in the ICU admitted patients compared to the non-ICU admitted patients.

**Table 2:** Baseline laboratory and imaging findings of patients admitted with COVID-19 infection (*N* = 1,560).

Characteristics	ICU admitted (*n* = 780)	Non-ICU admitted (*n* = 780)	*p*-value
Laboratory findings at admission, median (IQR) White Blood Cells [x103/μL] Hemoglobin [g/dl] Platelet count [x103/μL] Absolute neutrophil count [x103/μL] Lymphocytes [x103/μL] Prothrombin time [sec]^a^ INR^a^ APTT [sec] ^a^ D-Dimer [mg/L] ^a^ Fibrinogen [g/L] ^a^ Serum creatinine [μmol/L] Albumin [g/L] Alanine aminotransferase [U/L] Aspartate transaminase [U/L] NT-ProBNP [pg/ml] ^a^ Troponin-T HS [ng/L] ^a^ C-reactive protein [mg/L] ^a^ Procalcitonin [ng/ml] ^a^ Lactic acid [mmol/l] ^a^ Lactate dehydrogenase [U/L] ^a^ Ferritin [μg/L] ^a^ Creatinine kinase [U/L] ^a^ HbA1c % ^a^ Cholesterol [mmol/L] ^a^ Triglyceride [mmol/L] ^a^ High density lipoprotein [mmol/L] ^a^ Low density lipoprotein [mmol/L] ^a^	7.5 (4.6) 13.6 (2.4) 211 (101) 5.8 (4.2) 1 (0.6) 13 (2.6) 1.1 (0.2) 32.1 (6) 0.87 (1.4) 5.9 (3) 88 (35) 33 (8) 35.4 (32) 48 (40) 277.8 (987.7) 13 (31) 103.8 (133.6) 0.365 (0.8) 1.65 (1) 475.5 (230) 793 (921) 255 (464) 6.9 (2.7) 3.82 (1.7) 1.8 (1) 0.8 (0.5) 2.1 (2)	6.4 (2.9) 14.3 (2) 227 (108) 3.9 (2.6) 1.5 (1) 12.15 (2.7) 1.1 (0.2) 31.8 (5.4) 0.56 (0.7) 5 (2) 82 (21) 38 (6) 30 (24) 28 (23) 81 (732.2) 8 (6) 25.75 (59) 0.12 (0.2) 1.5 (0.9) 293 (164) 441 (498) 95 (103) 6.8 (2.4) 4.24 (1.7) 1.4 (1) 1 (0.4) 2.4 (1)	< 0.001^§^ < 0.001^§^ < 0.001^§^ < 0.001^§^ < 0.001^§^ < 0.001^§^ < 0.001^§^ 0.395^§^ < 0.001^§^ 0.161^§^ < 0.001^§^ < 0.001^§^ < 0.001^§^ < 0.001^§^ < 0.001^§^ < 0.001^§^ < 0.001^§^ < 0.001^§^ 0.013^§^ < 0.001^§^ < 0.001^§^ < 0.001^§^ 0.151^§^ 0.003^§^ < 0.001^§^ < 0.001^§^ 0.004^§^
Imaging studies at admission X-ray, *n* (%) ^b^ Clear Ground glass opacity Consolidation Infiltrates Interstitial abnormalities Patchy Opacity Pleural effusion Others Direction of abnormality Bilateral Right Lung Left lung Computed tomography, *n* (%) ^c^ Clear Ground glass opacity Consolidation Infiltrates Interstitial abnormalities Patchy Opacity Pleural effusion Pulmonary Embolism Others Direction of abnormality Bilateral Right Lung Left lung	134 (17.2) 110 (14.1) 154 (19.8) 238 (30.6) 14 (1.8) 248 (31.8) 23 (3.0) 33 (4.2) 570 (73.2) 38 (4.9) 37 (4.7) 7 (4.5) 120 (77.4) 78 (50.3) 10 (6.5) 32 (20.6) 36 (23.2) 27 (17.4) 14 (9.0) 40 (26) 139 (89.7) 3 (1.9) 6 (3.9)	327 (42.2) 65 (8.4) 66 (8.5) 165 (21.3) 26 (3.4) 177 (22.8) 11 (1.4) 32 (4.1) 312 (40.3) 81 (10.5) 55 (7.1) 2 (10) 14 (70) 5 (25) 3 (15) 6 (30) 2 (10) 1 (5.0) 0 10 (50) 16 (80) 2 (10) 0	<0.001^¶^ <0.001^¶^ <0.001^¶^ <0.001^¶^ 0.005^¶^ <0.001^¶^ 0.039^¶^ 0.205^¶^ <0.001^¶^ 0.296^¶^ 0.461^¶^ 0.033^¶^ 0.170^¶^ 0.424^¶^ 0.177^¶^ 0.154^¶^ 0.161^¶^ <0.001^¶^ 0.110^¶^

^§^ *p*-value obtained using Mann–Whitney test; ^¶^*p*-value obtained using Chi-square test; ICU, intensive care unit; INR, international normalized ratio; APTT, activated partial thromboplastin time; NT-proBNP, N-terminal-pro hormone BNP; HbA1c, hemoglobin A1c; ^a^ have missing data; ^b^ 1 patient did not do an x-ray in ICU group and 5 in the non-ICU group; ^c^ 625 patients did not do CT in ICU group and 760 patients in the non-ICU group.

There were significantly higher rates of abnormal X-ray and CT studies in ICU-admitted patients than non-ICU patients. About 73.1 and 89.7% of the ICU admitted patients had bilateral abnormalities in X-ray and CT scan, respectively. The prevalence of patients with a chest radiograph showing ground-glass opacity, consolidation, infiltrates, patchy opacity, pleural effusion, or interstitial abnormalities were more commonly seen in the ICU admitted patients (Table 2).

Also, the co-infections rate was higher in the ICU group. The most common co-infection in the ICU group was bacterial (31.7%) (Table 3). In terms of medications, a significantly higher proportion of patients were prescribed antiviral medications, tocilizumab, plasma protein fraction, methylprednisolone, and vasopressors (all with value of *p* <0.001) as shown in Table 3.

**Table 3:** Patient therapy and clinical outcome of patients admitted with COVID-19 diagnosis in Qatar (*N* = 1,560).

	ICU admitted (*n* = 780)	Non-ICU admitted (*n* = 780)	*p*-value^¶^
Medications during hospitalization, *n* (%) Azithromycin Hydroxychloroquine Lopinavir/ritonavir Darunavir/cobicistat Tocilizumab Ribavirin Plasma protein fraction Oseltamivir ACEI/ARB NSAID Methylprednisolone Vasopressor	761 (97.6%) 750 (96.2%) 381 (48.8%) 55 (7.1%) 514 (65.9%) 90 (11.5%) 132 (16.9%) 615 (78.8%) 174 (22.3%) 50 (6.4%) 615 (78.8%) 134 (17.2%)	622 (79.7%) 674 (86.4%) 137 (17.6%) 25 (3.2%) 4 (0.5%) 5 (0.6%) 0 521 (66.8%) 133 (17.1%) 24 (3.1%) 6 (0.8%) 0	<0.001 <0.001 <0.001 0.001 <0.001 <0.001 <0.001 <0.001 0.009 <0.001 <0.001 <0.001
In hospital co-infection, *n* (%) Bacterial Viral Fungal	319 (40.9%) 247 (31.7%) 10 (1.3%) 199 (25.5%)	20 (2.6%) 16 (2.1%) 3 (0.4%) 1 (0.1%)	<0.001 <0.001 0.051 <0.001
Length of stay in hospital [days], median (IQR)	22 (15)	5 (7)	<0.001^§^
Length of stay in ICU *[days]*, median (IQR)	8 (9)	NA	
Clinical Outcome, *n* (%) Discharged from ICU and remained in Hospital Discharged Home Still in the ICU Died	29 (3.7%) 636 (81.5%) 9 (1.2%) 106 (13.6%)	0 780 (100%) 0 0	<0.001

^§^*p*-value obtained using Mann–Whitney test; ^¶^*p*-value obtained using Chi-square test; ICU, intensive care unit; NSAID, Non-steroidal anti-inflammatory drugs; ACEI, Angiotensin-converting-enzyme inhibitors; ARB, Angiotensin II Receptor Blockers; NA, Not applicable.

### 3.2 Patient disposition

All patients in the non-ICU admitted group were discharged at the end of follow up. However, for the ICU admitted group, 81.5% of patients were discharged, 13.6% died, 3.7% were transferred to secondary care and 1.7% remained in ICU care by the end of the follow up. Longer hospital stay was observed in the ICU group [median (IQR) 22 (15) days vs. 5 (7) days, value of *p* < 0.001] (Table 3).

During hospitalization, 50.3% of ICU admitted patients required intubation and 2.9% needed extracorporeal membrane oxygenation support (Table 4). The most common reason for ICU admission was desaturation (45.5%), followed by acute respiratory distress syndrome (30.3%).

**Table 4:** COVID-19 patients admitted to ICU.

ICU parameters	*n* (%)
ICU course, *n* (%) Intubation Prone Position Extracorporeal membrane oxygenation (ECMO)	392 (50.3%) 456 (58.5%) 23 (2.9%)
Reason for ICU admission, *n* (%) Desaturation Acute respiratory distress syndrome Hypotension requiring resuscitation Tachypnoea ST-Elevation Myocardial Infarction Non-ST-Elevation Myocardial Infarction Diabetic ketoacidosis Stroke Cardiac arrest Venous thromboembolism Shock Others No documentation	355 (45.5%) 236 (30.3%) 10 (1.3%) 32 (4.1%) 30 (3.8%) 8 (1.0%) 14 (1.8%) 10 (1.3%) 4 (0.5%) 4 (0.5%) 8 (1.0%) 57 (7.3%) 12 (1.5%)

### 3.3 Risk factors for ICU admission and mortality

A univariate logistic regression analysis was conducted initially to identify the predictors for ICU admission among the patients diagnosed with COVID-19 (Supplementary material).

**Figure 1:**
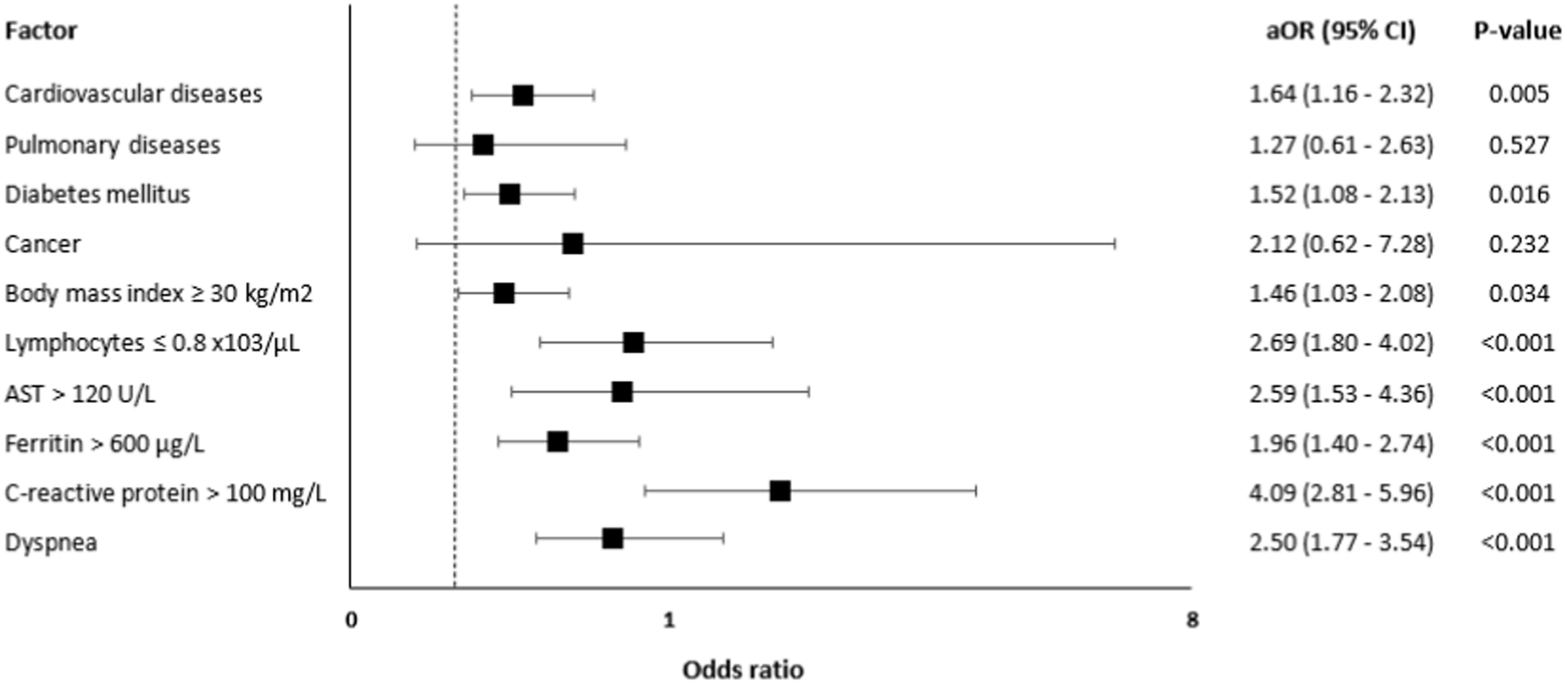
Risk factors for ICU admission among patients with COVID-19 (*N* = 1,560).

A multivariate logistic regression analysis (Figure 1) showed that independent predictors for ICU admission were CVD [aOR = 1.64, 95% CI: 1.16–2.32, value of *p* = 0.005], diabetes [aOR = 1.52, 95% CI: 1.08–2.13, value of *p* = 0.016], BMI ≥30 kg/m^2^ [aOR = 1.46, 95% CI: 1.03–2.08, value of *p* = 0.034], lymphocytes ≤0.8 ×103/μL [aOR = 2.69, 95% CI: 1.80–4.02, value of *p* <0.001], AST >120 U/L [aOR = 2.59, 95% CI: 1.53–4.36, value of *p* <0.001], ferritin >600 μg/L [aOR = 1.96, 95% CI: 1.40–2.74, value of *p* <0.001], CRP >100 mg/L [aOR = 4.09, 95% CI: 2.81–5.96, value of *p* <0.001], and dyspnea [aOR = 2.50, 95% CI: 1.77–3.54, value of *p* < 0.001].

**Figure 2:**
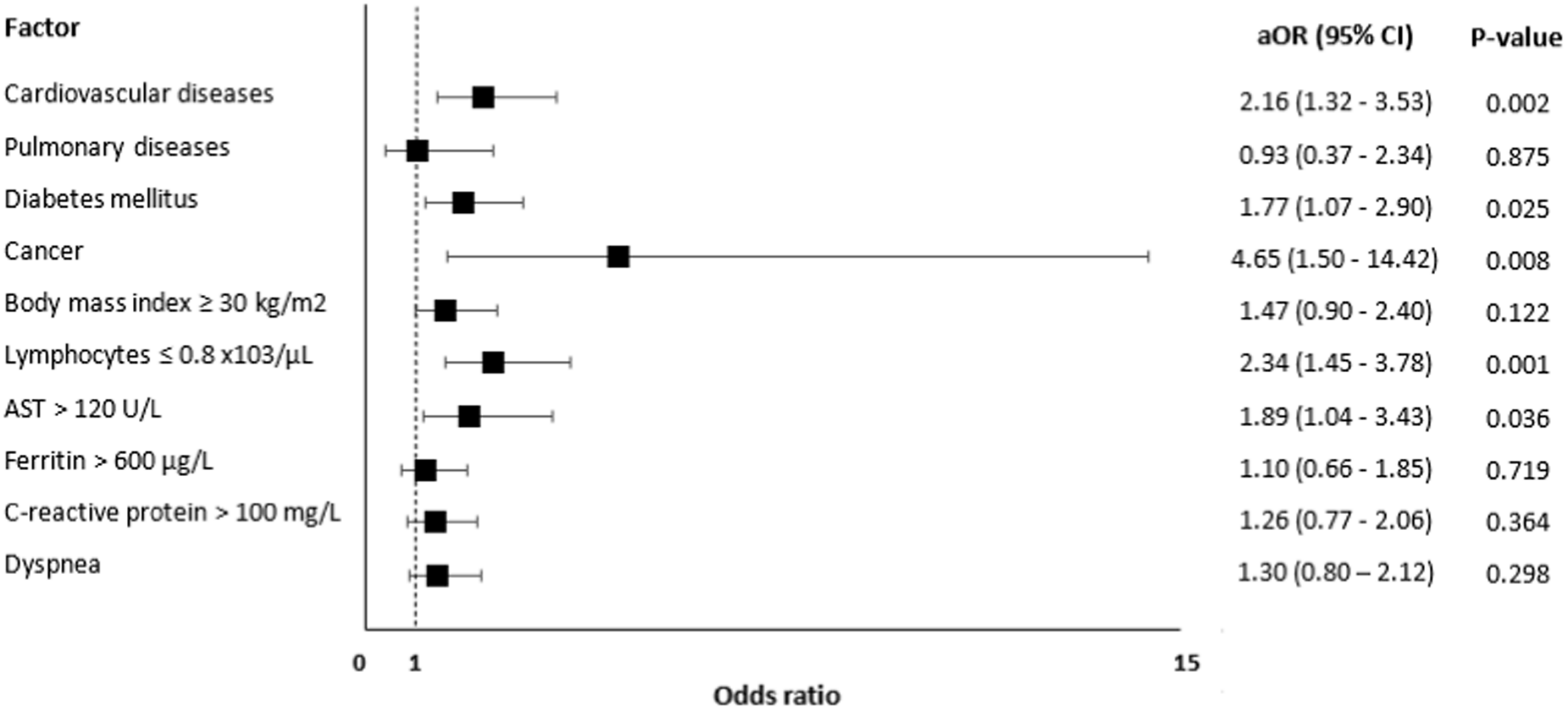
Risk factors for mortality among patients with COVID-19 (*N* = 1,560).

Risk factors associated with mortality are presented in Figure 2 and these include CVD [aOR=; 95% CI: 1.32–3.53, value of *p* = 0.002], diabetes [aOR = 1.77, 95% CI: 1.07–2.90, value of *p* = 0.025], cancer [aOR = 4.65, 95% CI: 1.50–14.42, value of *p* = 0.008], lymphocytes ≤0.8 x,103/μL [aOR = 2.34, 95% CI: 1.45–3.78, value of *p* = 0.001], and AST > 120 U/L [aOR = 1.89, 95% CI: 1.04–3.43, value of *p* = 0.036].

## 4 Discussion

This retrospective case–control study has recognized that advanced age and the male gender is an established risk factor for severe disease and was therefore matched (31, 36–38); thus, emphasizing other variables to be investigated in the study. The aim was early identification [as early as 24 h from admission] of the predictors and risk factors leading to ICU admission and in-hospital mortality. The study identified cardiovascular diseases, diabetes, obesity, dyspnea, lymphopenia, elevated ferritin, AST, and CRP as significant predictors and risk factors of ICU admission. In particular, cardiovascular diseases, diabetes, lymphopenia, and elevated AST were independent risk factors of ICU admission and in-hospital mortality.

The findings are consistent with several studies that reported a strong association between CVD and ICU admission and mortality (17, 22, 31, 37, 39–44). He F., et al. (39) reported that patients with CVD are more likely to deteriorate to severe disease, be admitted to the ICU, and require respiratory support. Moreover, these findings were also confirmed in a meta-analysis that reported that CVD is significantly associated with ICU admission and mortality (45). It is suggested that the association might be due to the cytokine storm leading to myocardial injury in these patients (46, 47). Moreover, the increase in cardiac demand in light of the underlying hypoxia caused by the infection can also lead to acute myocardial injury and exacerbation of stable heart failure (47, 48). Therefore, special care with frequent and heightened monitoring for the possible occurrence of acute cardiac events is highly recommended in this setting.

Furthermore, the current study found that diabetes is also an independent risk factor for ICU admission and mortality. This concurs with previous cohort studies’ results confirming the association of diabetes with poorer outcomes in patients diagnosed with COVID-19 (22, 31, 33, 37, 49, 50). Suggested plausible association of the increased susceptibility to the development of severe or even critical COVID-19 infection in diabetic patients include higher affinity cellular binding and efficient virus entry, decreased viral clearance, diminished T-cell function, and increased susceptibility to hyperinflammation and cytokine storm syndrome (51, 52).

On the other hand, obesity was associated with an increased risk of ICU admission, but not mortality. This is consistent with the findings of two previous studies (33, 53). Similar outcomes have also been observed in France, demonstrating that a BMI ≥ 35 kg/m^2^ is an independent risk factor of severity in COVID-19 infection (54). Furthermore, considering the previous two points, a local study conducted by Omarni et al. describing the first 5,000 cases of COVID-19 in Qatar reported that obesity and diabetes were associated with ICU admission, but not death (55). However, the study only included 108 ICU patients, which required confirmation in large-scale research.

In addition, the present study has revealed that cancer is associated with a four-fold increase in the risk of death. However, there are conflicting results in the literature, where some studies found an increased risk of poor prognosis among COVID-19 patients with cancer while other studies failed to demonstrate such an association (56–60). One suggested hypothesis for this association is the downregulation of the immune response in cancer patients resulting in diminishing cytokine storms and thus the reduction in the severity of the infection (61, 62). However, a recent meta-analysis of 32 studies revealed that cancer was associated with poorer clinical outcomes among patients with COVID-19 (63).

Lymphopenia and high AST were associated with an around two-fold increase in the risk for ICU admission and the risk of death. High ferritin and CRP levels were also found to be predictors for ICU admission, with CRP having a four-fold increase in the risk. In their meta-analysis, Huang et al. reported that lower lymphocyte count was seen in patients who died, experienced acute respiratory distress syndrome, and had severe COVID-19 infection (64). Moreover, consistent with the present study, another meta-analysis reported that lymphopenia, high ferritin, CRP, and AST are strong predictors of severe disease (65). Similarly, a study conducted by Wang et al. reported a positive correlation between CRP levels and the extent of lung lesions and disease severity (66).

Fever, dry cough, and dyspnea were the most common symptoms in ICU admitted patients. This study found that dyspnea is a predictor of ICU admission. Previous studies assessing clinical symptoms associated with disease outcomes reported that dyspnea is an independent predictor for in-hospital death (21, 67–70). In one meta-analysis, dyspnea was the only significant symptom associated with COVID-19 disease severity and ICU admission: 6.6-fold increased risk of ICU admission (71). Considering the significance of dyspnea in predicting ICU admission, it is essential to enhance public awareness of this symptom and perform close monitoring of patients with dyspnea in an outpatient setting.

COVID-19, especially severe disease, was found to be associated with an increased risk of pulmonary embolism, with a reported prevalence of 32% and up to 49% among critically ill patients (72). However, in our case–control study, pulmonary embolism was diagnosed in 9% only of the ICU patients, which is considered relatively low. This could be because our study was conducted early in the COVID-19 pandemic when the association of COVID-19 with thromboembolism was not yet confirmed; in addition, a systemic diagnostic approach to detect thrombotic events was not applied in our study in view of the retrospective nature of the study.

This study has some limitations that should be considered. First, due to this study’s retrospective nature, smoking status, and some laboratory findings (such as D-Dimer, prothrombin, INR, APTT, fibrinogen, troponin I, and N-terminal pro-brain natriuretic peptide) were not documented in many patients. Therefore, this has limited our ability to analyze these variables. Second, although our patient population was diverse, this study was done in a single geographic region. Therefore, large-scale cohort studies involving multiple nations are needed to support our findings.

## 5 Conclusion

In conclusion, the current study investigating the early predictors of ICU admission and mortality in patients diagnosed with COVID-19 has revealed that CVD, diabetes, lymphopenia, and increased AST are independent predictors for ICU admission and in-hospital death. Obesity, ferritin, and CRP levels are associated with an increased risk of ICU admission, while cancer is a strong predictor of mortality. Health care systems and clinicians should consider these early predictors of severe COVID-19 when triaging patients to facilitate early medical intervention, close monitoring, and better therapeutic outcomes.

## Data availability statement

The raw data supporting the conclusions of this article will be made available by the authors, without undue reservation.

## Ethics statement

The studies involving humans were approved by Hamad Medical Corporation, Medical Research Center, Study ID #MRC-01-20-338. The studies were conducted in accordance with the local legislation and institutional requirements. The ethics committee/institutional review board waived the requirement of written informed consent for participation from the participants or the participants’ legal guardians/next of kin because the IRB has determined and documented at a convened meeting that the research involves no greater than Minimal Risk and that no additional risks have been identified.

## Author contributions

SAb: Conceptualization, Data curation, Formal analysis, Investigation, Methodology, Project administration, Supervision, Writing – original draft, Writing – review & editing. SAl: Conceptualization, Data curation, Investigation, Methodology, Writing – original draft, Writing – review & editing. NO: Conceptualization, Data curation, Funding acquisition, Investigation, Methodology, Validation, Writing – original draft, Writing – review & editing. RE: Data curation, Methodology, Validation, Writing – review & editing. EE: Data curation, Methodology, Validation, Writing – review & editing. EA-A: Data curation, Methodology, Validation, Writing – review & editing. RB: Data curation, Methodology, Validation, Writing – review & editing. RG: Data curation, Methodology, Validation, Writing – review & editing. AR: Data curation, Formal analysis, Investigation, Methodology, Software, Validation, Writing – review & editing. FH: Data curation, Methodology, Validation, Writing – review & editing. MA-A: Data curation, Methodology, Validation, Writing – review & editing. AKara: Data curation, Investigation, Methodology, Software, Validation, Writing – review & editing. FA: Data curation, Methodology, Validation, Writing – review & editing. RA: Data curation, Methodology, Validation, Writing – review & editing. AKard: Data curation, Methodology, Validation, Writing – review & editing. AA: Methodology, Validation, Visualization, Writing – review & editing. AA-A: Investigation, Software, Validation, Visualization, Writing – review & editing. MK: Investigation, Validation, Visualization, Writing – review & editing. MA: Investigation, Validation, Visualization, Writing – review & editing. MA-H: Investigation, Project administration, Supervision, Validation, Visualization, Writing – review & editing.

## Glossary

COVID-19: Coronavirus disease 2019
WHO: World Health Organization
ICU: Intensive care unit
BMI: Body mass index
HMC: Hamad Medical Corporation
RT-PCR: Positive real-time polymerase chain reaction
CVD: Cardiovascular disease
CRP: C-reactive protein
ALT: Alanine aminotransferase
AST: Aspartate aminotransferase
INR: International normalized ratio
LDH: Lactate dehydrogenase
NT Pro-BNP: N-terminal pro-brain natriuretic peptide
CK: Creatinine kinase
SARS: Severe acute respiratory syndrome
MERS: Middle East Respiratory Syndrome
SARS-CoV: SARS-associated coronavirus
IQR: Interquartile range
OR: Crude odds ratio
aOR: Adjusted odds ratio
CCI: Charlson comorbidity index
HTN: Hypertension
CAD: Coronary artery disease
HF: Heart Failure
NSAID: Non-steroidal anti-inflammatory drugs
ACEI: Angiotensin-converting-enzyme inhibitors
ARB: Angiotensin II Receptor
APTT: Activated partial thromboplastin time
HbA1c: Hemoglobin A1c
