# Supplementary material for: Early predictors of intensive care unit admission among COVID-19 patients in Qatar
Source: Front Public Health. 2024 Mar 20;12:1278046. doi: 10.3389/fpubh.2024.1278046 (PMC10987715; doi:10.3389/fpubh.2024.1278046)
Supplement: Supplementary file 1 [file Data_Sheet_1.DOCX]

Supplementary material:

Univariate analysis of ICU admission risk factors in patients admitted with COVID-19 infection

| **Characteristic** | **ICU**  **(n=780), n(%)** | **Non-ICU  (n=780), n(%)** | **Univariate** | | **P-Value** |
| --- | --- | --- | --- | --- | --- |
|  |  |  | **OR** | **95% CI** |  |
| **Comorbidities** |  |  | 1.82  1.53  1.43  2.15  2.46  2.70  2.01  1.86  0.50  4.08  2.87  0.30 | 1.48 – 2.24  1.25 – 1.89  1.16 – 1.76  1.46 – 3.16  1.02 – 5.97  1.65 – 4.41  0.50 – 8.06  1.14 – 3.03  0.19 – 1.33  1.52 – 10.92  1.13 – 7.33  0.12 – 0.74 |  |
| Diabetes mellitus | 349 (44.7) | 240 (30.8) |  |  | <0.001 |
| Cardiovascular diseases (CAD, HTN, HF) | 323 (41.4) | 246 (31.5) |  |  | <0.001 |
| Hypertension (HTN) | 296 (37.9) | 234 (30) |  |  | <0.001 |
| Coronary artery disease (CAD) | 83 (10.6) | 41 (5.3) |  |  | <0.001 |
| Heart Failure (HF) | 17 (2.2) | 7 (0.9) |  |  | 0.046 |
| Chronic kidney disease | 59 (7.6) | 23 (2.9) |  |  | <0.001 |
| Chronic Liver diseases | 6 (0.8) | 3 (0.4) |  |  | 0.326 |
| Pulmonary diseases | 47 (6.0) | 26 (3.3) |  |  | 0.013 |
| Peripheral Vascular Disease | 6 (0.8) | 12 (1.5) |  |  | 0.163 |
| Immunosuppression | 20 (2.6) | 5 (0.6) |  |  | 0.005 |
| Stroke | 17 (2.2) | 6 (0.8) |  |  | 0.027 |
| Cancer | 20 (2.6) | 6 (0.8) |  |  | 0.006 |
| **Charlson score (≥ 3)** | 93 (11.9) | 54 (6.9) | 1.82 | 1.28 – 2.59 | <0.001 |
| **Body mass index (≥ 30 kg/m^2^)** | 251 (32.4) | 173 (26.7) | 1.32 | 1.045 – 1.66 | 0.020 |
| **Symptoms at admission**  Asymptomatic  Dyspnea  Fever  Dry cough  Productive cough  Hemoptysis  Abdominal Pain  Nausea Vomiting Chest pain Fatigue Myalgia Headache Confusion Sore throat Diarrhea | 24 (3.1) 404 (51.8) 635 (81.4) 481 (61.7) 87 (11.2) 10 (1.3) 45 (5.8) 43 (5.5) 69 (8.8) 98 (12.6) 102 (13.1) 186 (23.8) 74 (9.5) 13 (1.7) 105 (13.5) 48 (6.2) | 162 (20.8) 134 (17.2) 507 (65) 440 (56.4) 23 (2.9) 3 (0.4) 20 (2.6) 14 (1.8) 27 (3.5) 33 (4.2) 37 (4.7) 161 (20.6) 77 (9.9) 5 (0.6) 126 (16.2) 28 (3.6) | 0.12  5.18  2.36  1.24  4.13  3.36  2.33  3.19  2.71  3.25  3.02  1.20  0.96  2.63  0.81  1.77 | 0.08 – 0.19  4.10 – 6.54  1.87 – 2.98  1.02 – 1.52  2.58 – 6.62  0.92 – 12.27  1.36 – 3.98  1.73 – 5.88  1.71 – 4.27  2.16 – 4.89  2.05 – 4.46  0.95 – 1.53  0.68 – 1.34  0.93 – 7.41  0.61 – 1.07  1.09 – 2.84 | <0.001  <0.001  <0.001  0.035  <0.001  0.066  0.002  <0.001  <0.001  <0.001  <0.001  0.128  0.797  0.068  0.135  0.020 |
| **Vital signs at admission**  Mean arterial pressure [ < 65 mmHg]  Heart rate [ ≥ 100 bpm]  Temperature [ ≥ 39 ^0^C]  Respiratory rate [≥ 24 rate/min]  Oxygen Saturation [ ≤ 88%] | 29 (3.7)  331 (42.4)  104 (13.3)  343 (44)  95 (12.2) | 1 (0.1)  225 (28.8)  45 (5.8)  31 (4.0)  1 (0.1) | 30.08  1.82  2.51  18.96  108 | 4.09 – 221.38  1.47 – 2.24  1.74 – 3.62  12.89 – 27.90  15 – 777 | <0.001  <0.001  <0.001  <0.001  <0.001 |
| **Laboratory findings at admission**  White Blood Cells [ > 10 x103/μL]  Haemoglobin [ ≤ 10 g/dl]  Platelet count [ > 400 x103/μL]  ANC [ > 10.6 x103/μL]  Lymphocytes [ ≤ 0.8 x103/μL]  D-Dimer [ ≥ 0.5 mg/L]  Fibrinogen [ > 4.1 g/L]  Serum creatinine [ ≥ 133 μmol/L]  Albumin [ < 30 g/L]  ALT [ > 120 U/L]  AST [ > 120 U/L]  NT-ProBNP [ >450 pg/ml]  Troponin-T HS [ ≥ 50 ng/L]  C-reactive protein [> 100 mg/L]  Procalcitonin [ > 1 ng/ml]  Lactic acid [ > 2.2 mmol/l]  Lactate dehydrogenase [ > 390 U/L]  Ferritin [ >600 μg/L] | 195 (25)  49 (6.3)  33 (4.2)  112 (14.5)  261 (33.8)  445 (71.8)  458 (81.1)  109 (14)  528 (67.9)  93 (12)  160 (20.8)  121 (39.4)  96 (20.7)  395 (51.7)  128 (23.5)  76 (22.6)  408 (70.1)  407 (60.2) | 62 (8.0)  8 (1.0)  38 (4.9)  14 (1.8)  83 (10.7)  88 (47.6)  9 (69.2)  26 (3.3)  216 (27.9)  53 (6.9)  43 (5.7)  12 (25.5)  8 (3.7)  88 (11.8)  8 (12.1)  45 (17.8)  35 (22.3)  127 (33.5) | 3.84  6.44  0.86  9.22  4.27  2.80  1.90  4.71  5.47  1.83  4.34  1.90  6.90  8.02  2.23  1.35  8.17  3.00 | 2.83 – 5.22  3.03 – 13.70  0.53 – 1.39  5.24 – 16.23  3.25 – 5.60  2.00 – 3.93  0.58 – 6.29  3.03 – 7.32  4.40 – 6.80  1.28 – 2.60  3.05 – 6.18  0.95 – 3.80  3.29 – 14.47  6.16 - 10.44  1.04 – 4.78  0.90 – 2.04  5.40 – 12.39  2.31 – 3.91 | <0.001  <0.001  0.537  <0.001  <0.001  <0.001  0.292  <0.001  <0.001  <0.001  <0.001  0.071  <0.001  <0.001  0.040  0.152  <0.001  <0.001 |
| **X-ray at admission**  Clear  Ground glass opacity  Consolidation  Infiltrates  Patchy Opacity  Pleural effusion | 134 (17.2)  110 (14.1)  154 (19.8)  238 (30.6)  248 (31.8)  23 (3.0) | 327 (42.2)  65 (8.4)  66 (8.5)  165 (21.3)  177 (22.8)  11 (1.4) | 0.29  1.80  2.65  1.63  1.58  2.11 | 0.23 – 0.36  1.30 – 2.48  1.95 – 3.60  1.30 – 2.05  1.26 – 1.98  1.02 - 4.37 | <0.001  <0.001  <0.001  <0.001  <0.001  0.043 |
| ^OR: Odds ratio; CI: Confidence interval; ICU: intensive care until; ANC: Absolute neutrophil count; ALT: Alanine aminotransferase; AST: Aspartate transaminase; NT-proBNP: N-terminal-pro hormone BNP^ | | | | | |
